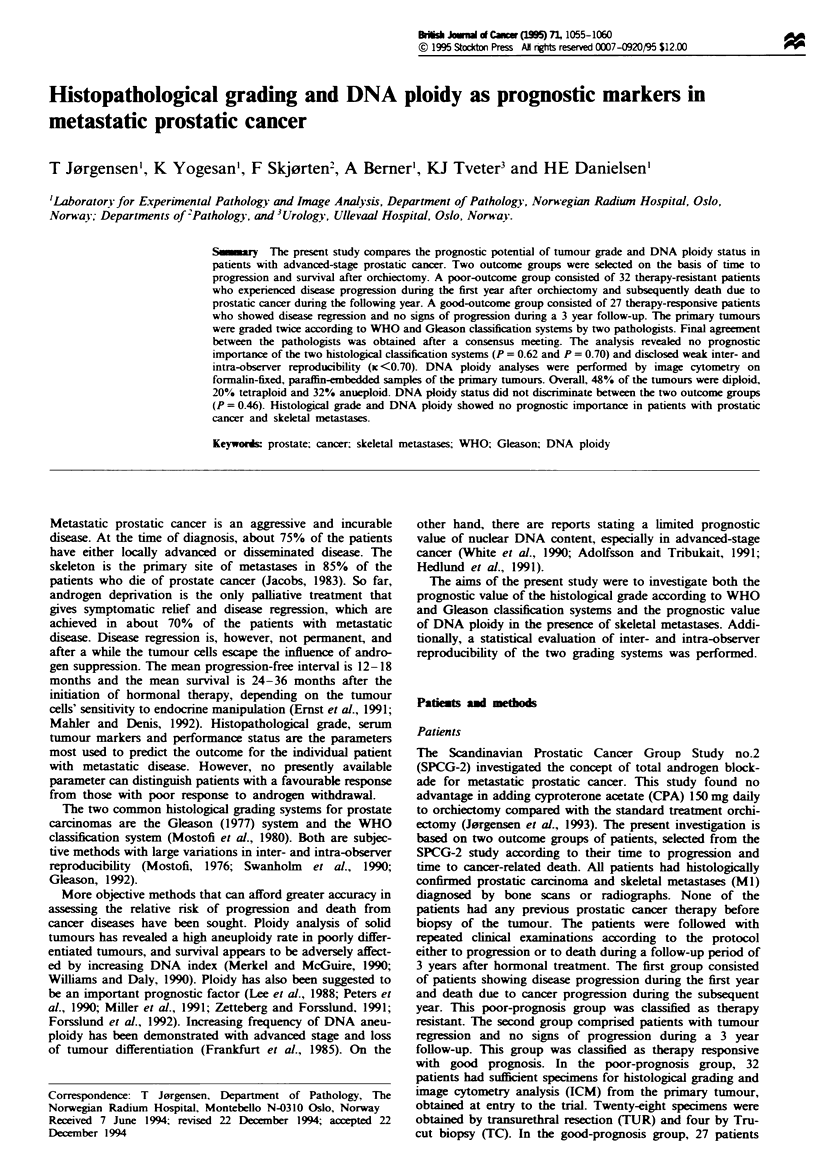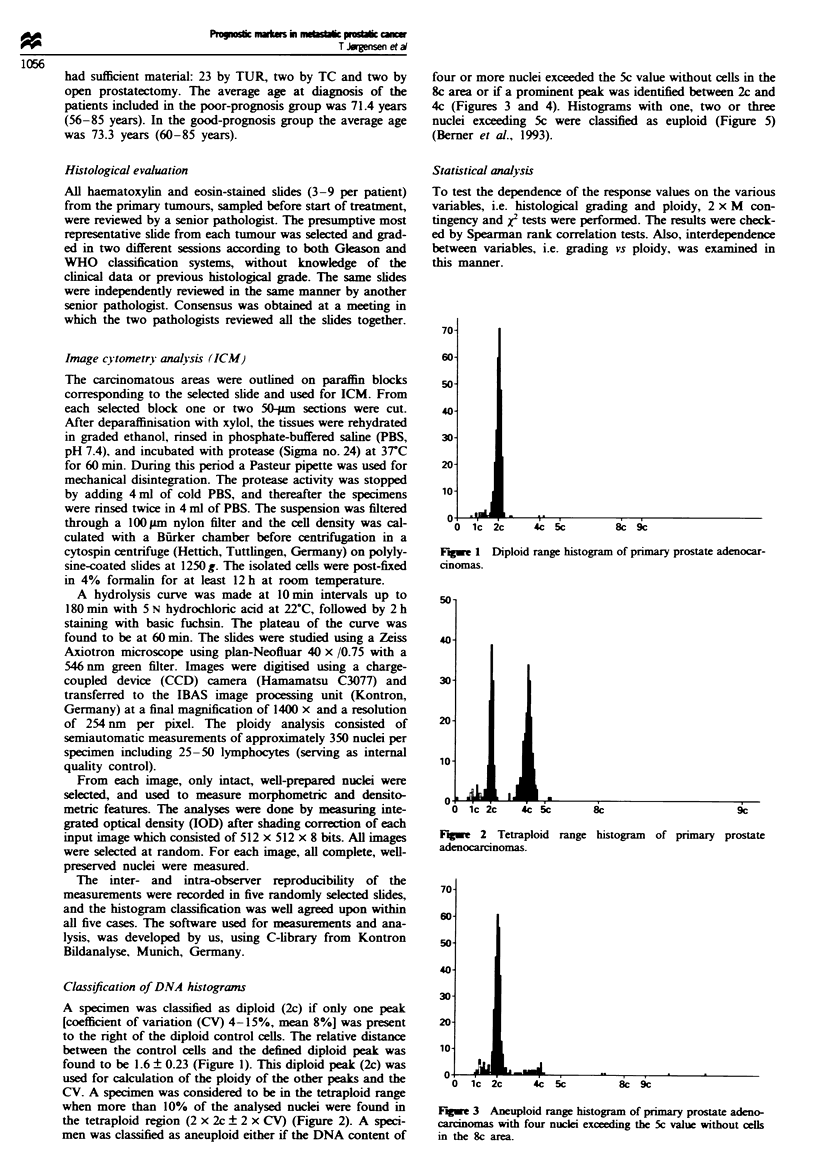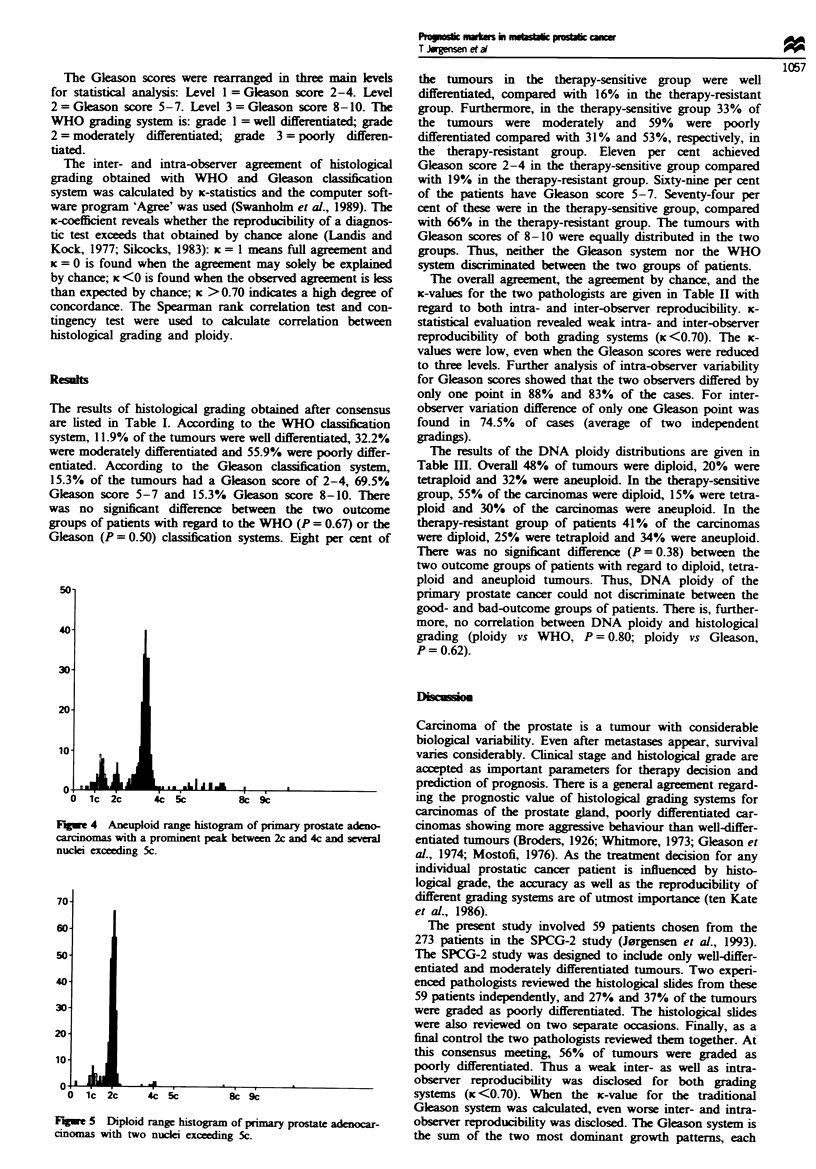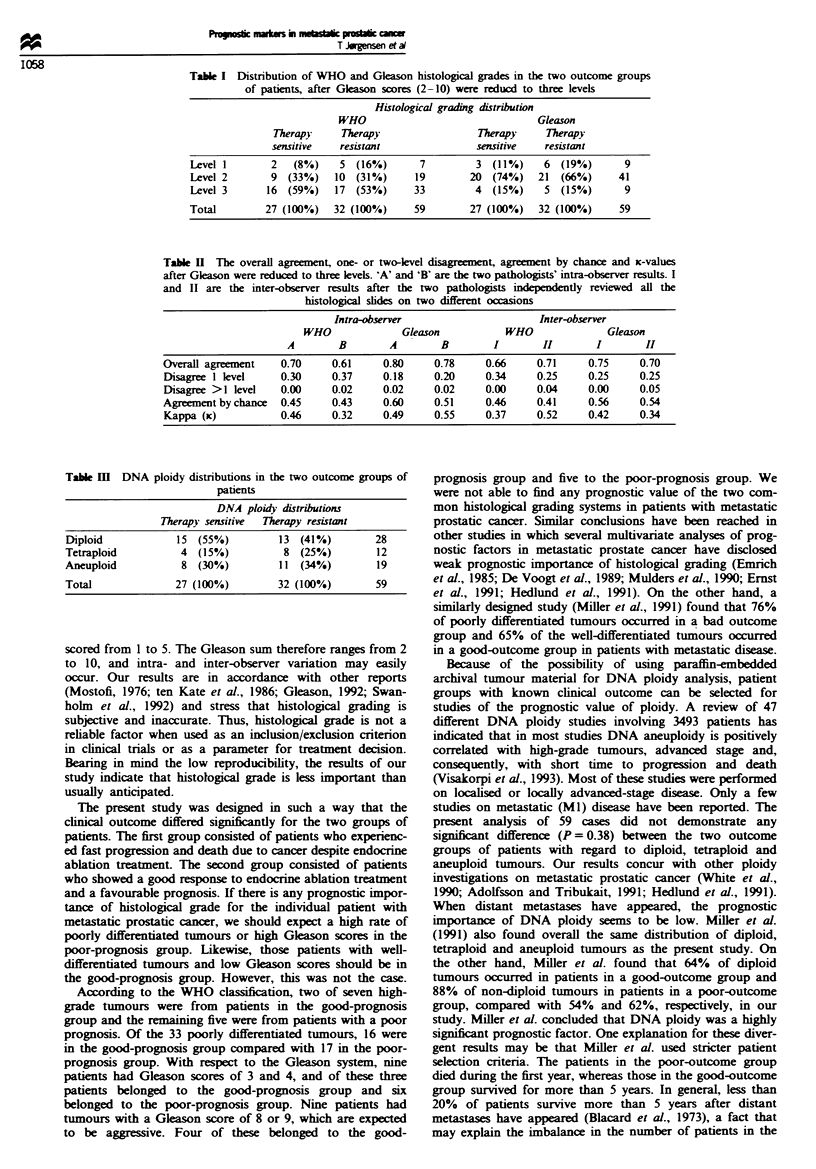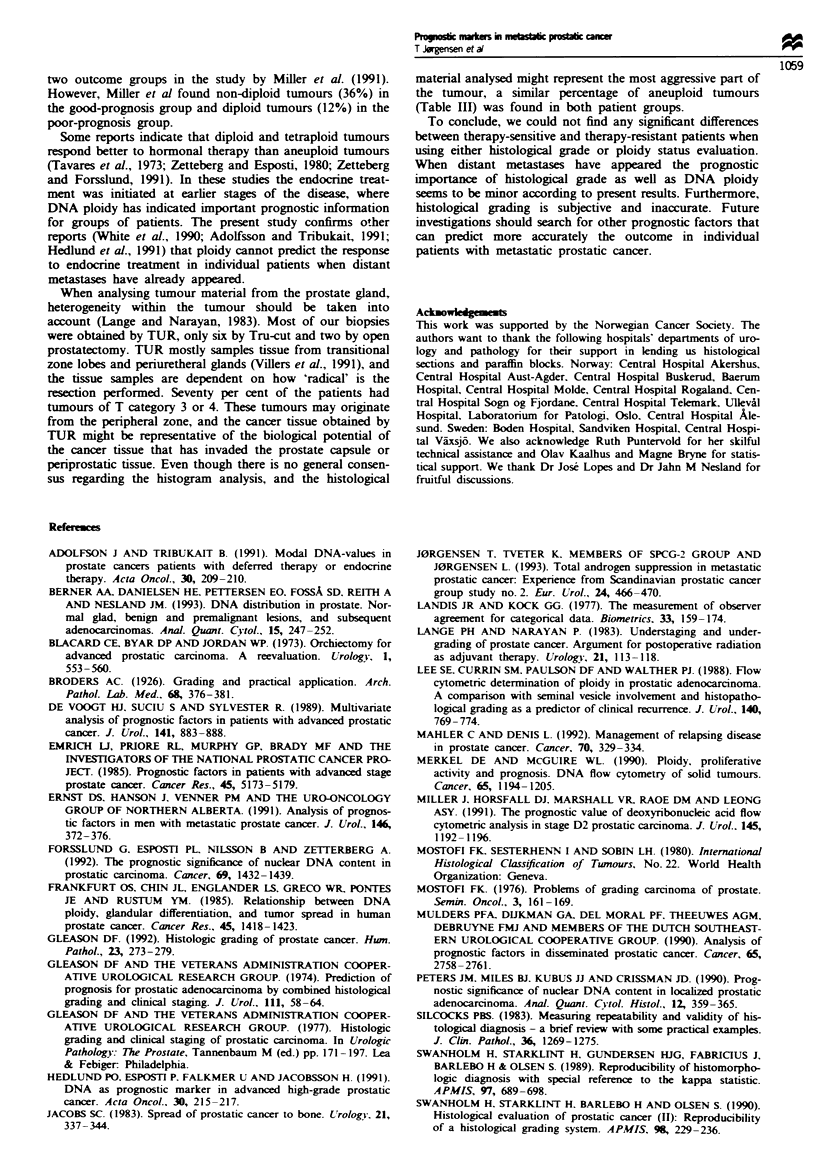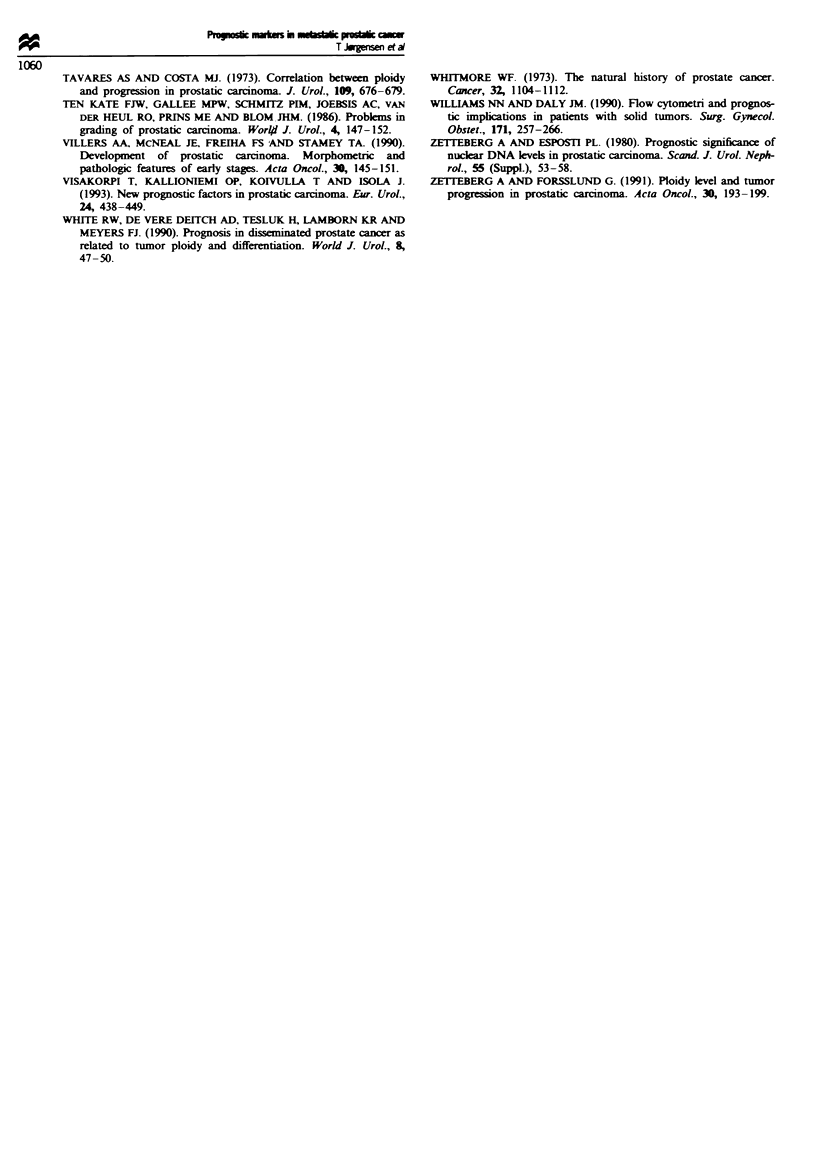# Histopathological grading and DNA ploidy as prognostic markers in metastatic prostatic cancer.

**DOI:** 10.1038/bjc.1995.203

**Published:** 1995-05

**Authors:** T. Jørgensen, K. Yogesan, F. Skjørten, A. Berner, K. J. Tveter, H. E. Danielsen

**Affiliations:** Department of Pathology, Norwegian Radium Hospital, Oslo.

## Abstract

The present study compares the prognostic potential of tumour grade and DNA ploidy status in patients with advanced-stage prostatic cancer. Two outcome groups were selected on the basis of time to progression and survival after orchiectomy. A poor-outcome group consisted of 32 therapy-resistant patients who experienced disease progression during the first year after orchiectomy and subsequently death due to prostatic cancer during the following year. A good-outcome group consisted of 27 therapy-responsive patients who showed disease regression and no signs of progression during a 3 year follow-up. The primary tumours were graded twice according to WHO and Gleason classification systems by two pathologists. Final agreement between the pathologists was obtained after a consensus meeting. The analysis revealed no prognostic importance of the two histological classification systems (P = 0.62 and P = 0.70) and disclosed weak inter- and intra-observer reproducibility (kappa < 0.70). DNA ploidy analyses were performed by image cytometry on formalin-fixed, paraffin-embedded samples of the primary tumours. Overall, 48% of the tumours were diploid, 20% tetraploid and 32% anueploid. DNA ploidy status did not discriminate between the two outcome groups (P = 0.46). Histological grade and DNA ploidy showed no prognostic importance in patients with prostatic cancer and skeletal metastases.


British Journal of Cancer (1995) 71, 1055-1060

© 1995 Stockton Press All rights reserved 0007-0920/95 $12.00

### Histopathological grading and DNA ploidy as prognostic markers in metastatic prostatic cancer

T Jørgensen[1], K Yogesan[1], F Skjørten[2], A Berner[1], KJ Tveter[3] and HE Danielsen[1]

[1]*Laboratory for Experimental Pathology and Image Analysis, Department of Pathology, Norwegian Radium Hospital, Oslo, Norway; Departments of* [2]*Pathology, and* [3]*Urology, Ullevaal Hospital, Oslo, Norway.*

**Summary** The present study compares the prognostic potential of tumour grade and DNA ploidy status in patients with advanced-stage prostatic cancer. Two outcome groups were selected on the basis of time to progression and survival after orchiectomy. A poor-outcome group consisted of 32 therapy-resistant patients who experienced disease progression during the first year after orchiectomy and subsequently death due to prostatic cancer during the following year. A good-outcome group consisted of 27 therapy-responsive patients who showed disease regression and no signs of progression during a 3 year follow-up. The primary tumours were graded twice according to WHO and Gleason classification systems by two pathologists. Final agreement between the pathologists was obtained after a consensus meeting. The analysis revealed no prognostic importance of the two histological classification systems ($P = 0.62$ and $P = 0.70$) and disclosed weak inter- and intra-observer reproducibility ($\kappa < 0.70$). DNA ploidy analyses were performed by image cytometry on formalin-fixed, paraffin-embedded samples of the primary tumours. Overall, 48% of the tumours were diploid, 20% tetraploid and 32% aneuploid. DNA ploidy status did not discriminate between the two outcome groups ($P = 0.46$). Histological grade and DNA ploidy showed no prognostic importance in patients with prostatic cancer and skeletal metastases.

**Keywords:** prostate; cancer; skeletal metastases; WHO; Gleason; DNA ploidy

Metastatic prostatic cancer is an aggressive and incurable disease. At the time of diagnosis, about 75% of the patients have either locally advanced or disseminated disease. The skeleton is the primary site of metastases in 85% of the patients who die of prostate cancer (Jacobs, 1983). So far, androgen deprivation is the only palliative treatment that gives symptomatic relief and disease regression, which are achieved in about 70% of the patients with metastatic disease. Disease regression is, however, not permanent, and after a while the tumour cells escape the influence of androgen suppression. The mean progression-free interval is 12-18 months and the mean survival is 24-36 months after the initiation of hormonal therapy, depending on the tumour cells' sensitivity to endocrine manipulation (Ernst *et al.*, 1991; Mahler and Denis, 1992). Histopathological grade, serum tumour markers and performance status are the parameters most used to predict the outcome for the individual patient with metastatic disease. However, no presently available parameter can distinguish patients with a favourable response from those with poor response to androgen withdrawal.

The two common histological grading systems for prostate carcinomas are the Gleason (1977) system and the WHO classification system (Mostofi *et al.*, 1980). Both are subjective methods with large variations in inter- and intra-observer reproducibility (Mostofi, 1976; Swanholm *et al.*, 1990; Gleason, 1992).

More objective methods that can afford greater accuracy in assessing the relative risk of progression and death from cancer diseases have been sought. Ploidy analysis of solid tumours has revealed a high aneuploidy rate in poorly differentiated tumours, and survival appears to be adversely affected by increasing DNA index (Merkel and McGuire, 1990; Williams and Daly, 1990). Ploidy has also been suggested to be an important prognostic factor (Lee *et al.*, 1988; Peters *et al.*, 1990; Miller *et al.*, 1991; Zetteberg and Forsslund, 1991; Forsslund *et al.*, 1992). Increasing frequency of DNA aneuploidy has been demonstrated with advanced stage and loss of tumour differentiation (Frankfurt *et al.*, 1985). On the other hand, there are reports stating a limited prognostic value of nuclear DNA content, especially in advanced-stage cancer (White *et al.*, 1990; Adolfsson and Tribukait, 1991; Hedlund *et al.*, 1991).

The aims of the present study were to investigate both the prognostic value of the histological grade according to WHO and Gleason classification systems and the prognostic value of DNA ploidy in the presence of skeletal metastases. Additionally, a statistical evaluation of inter- and intra-observer reproducibility of the two grading systems was performed.

### Patients and methods

### *Patients*

The Scandinavian Prostatic Cancer Group Study no.2 (SPCG-2) investigated the concept of total androgen blockade for metastatic prostatic cancer. This study found no advantage in adding cyproterone acetate (CPA) 150 mg daily to orchiectomy compared with the standard treatment orchiectomy (Jørgensen *et al.*, 1993). The present investigation is based on two outcome groups of patients, selected from the SPCG-2 study according to their time to progression and time to cancer-related death. All patients had histologically confirmed prostatic carcinoma and skeletal metastases (M1) diagnosed by bone scans or radiographs. None of the patients had any previous prostatic cancer therapy before biopsy of the tumour. The patients were followed with repeated clinical examinations according to the protocol either to progression or to death during a follow-up period of 3 years after hormonal treatment. The first group consisted of patients showing disease progression during the first year and death due to cancer progression during the subsequent year. This poor-prognosis group was classified as therapy resistant. The second group comprised patients with tumour regression and no signs of progression during a 3 year follow-up. This group was classified as therapy responsive with good prognosis. In the poor-prognosis group, 32 patients had sufficient specimens for histological grading and image cytometry analysis (ICM) from the primary tumour, obtained at entry to the trial. Twenty-eight specimens were obtained by transurethral resection (TUR) and four by Tru-cut biopsy (TC). In the good-prognosis group, 27 patients

Correspondence: T Jørgensen, Department of Pathology, The Norwegian Radium Hospital, Montebello N-0310 Oslo, Norway
Received 7 June 1994; revised 22 December 1994; accepted 22 December 1994

had sufficient material: 23 by TUR, two by TC and two by open prostatectomy. The average age at diagnosis of the patients included in the poor-prognosis group was 71.4 years (56–85 years). In the good-prognosis group the average age was 73.3 years (60–85 years).

### *Histological evaluation*

All haematoxylin and eosin-stained slides (3–9 per patient) from the primary tumours, sampled before start of treatment, were reviewed by a senior pathologist. The presumptive most representative slide from each tumour was selected and graded in two different sessions according to both Gleason and WHO classification systems, without knowledge of the clinical data or previous histological grade. The same slides were independently reviewed in the same manner by another senior pathologist. Consensus was obtained at a meeting in which the two pathologists reviewed all the slides together.

### *Image cytometry analysis (ICM)*

The carcinomatous areas were outlined on paraffin blocks corresponding to the selected slide and used for ICM. From each selected block one or two 50-μm sections were cut. After deparaffinisation with xylol, the tissues were rehydrated in graded ethanol, rinsed in phosphate-buffered saline (PBS, pH 7.4), and incubated with protease (Sigma no. 24) at 37°C for 60 min. During this period a Pasteur pipette was used for mechanical disintegration. The protease activity was stopped by adding 4 ml of cold PBS, and thereafter the specimens were rinsed twice in 4 ml of PBS. The suspension was filtered through a 100 μm nylon filter and the cell density was calculated with a Bürker chamber before centrifugation in a cytospin centrifuge (Hettich, Tuttlingen, Germany) on polylysine-coated slides at 1250 *g*. The isolated cells were post-fixed in 4% formalin for at least 12 h at room temperature.

A hydrolysis curve was made at 10 min intervals up to 180 min with 5 N hydrochloric acid at 22°C, followed by 2 h staining with basic fuchsin. The plateau of the curve was found to be at 60 min. The slides were studied using a Zeiss Axiotron microscope using plan-Neofluar 40 × /0.75 with a 546 nm green filter. Images were digitised using a charge-coupled device (CCD) camera (Hamamatsu C3077) and transferred to the IBAS image processing unit (Kontron, Germany) at a final magnification of 1400 × and a resolution of 254 nm per pixel. The ploidy analysis consisted of semiautomatic measurements of approximately 350 nuclei per specimen including 25–50 lymphocytes (serving as internal quality control).

From each image, only intact, well-prepared nuclei were selected, and used to measure morphometric and densitometric features. The analyses were done by measuring integrated optical density (IOD) after shading correction of each input image which consisted of 512 × 512 × 8 bits. All images were selected at random. For each image, all complete, well-preserved nuclei were measured.

The inter- and intra-observer reproducibility of the measurements were recorded in five randomly selected slides, and the histogram classification was well agreed upon within all five cases. The software used for measurements and analysis, was developed by us, using C-library from Kontron Bildanalyse, Munich, Germany.

### *Classification of DNA histograms*

A specimen was classified as diploid (2c) if only one peak [coefficient of variation (CV) 4–15%, mean 8%] was present to the right of the diploid control cells. The relative distance between the control cells and the defined diploid peak was found to be 1.6 ± 0.23 (Figure 1). This diploid peak (2c) was used for calculation of the ploidy of the other peaks and the CV. A specimen was considered to be in the tetraploid range when more than 10% of the analysed nuclei were found in the tetraploid region (2 × 2c ± 2 × CV) (Figure 2). A specimen was classified as aneuploid either if the DNA content of four or more nuclei exceeded the 5c value without cells in the 8c area or if a prominent peak was identified between 2c and 4c (Figures 3 and 4). Histograms with one, two or three nuclei exceeding 5c were classified as euploid (Figure 5) (Berner *et al.*, 1993).

### *Statistical analysis*

To test the dependence of the response values on the various variables, i.e. histological grading and ploidy, 2 × M contingency and $\chi^2$ tests were performed. The results were checked by Spearman rank correlation tests. Also, interdependence between variables, i.e. grading *vs* ploidy, was examined in this manner.

**Figure 1** Diploid range histogram of primary prostate adenocarcinomas.

**Figure 2** Tetraploid range histogram of primary prostate adenocarcinomas.

**Figure 3** Aneuploid range histogram of primary prostate adenocarcinomas with four nuclei exceeding the 5c value without cells in the 8c area.

The Gleason scores were rearranged in three main levels for statistical analysis: Level 1 = Gleason score 2–4. Level 2 = Gleason score 5–7. Level 3 = Gleason score 8–10. The WHO grading system is: grade 1 = well differentiated; grade 2 = moderately differentiated; grade 3 = poorly differentiated.

The inter- and intra-observer agreement of histological grading obtained with WHO and Gleason classification system was calculated by κ-statistics and the computer software program 'Agree' was used (Swanholm *et al.*, 1989). The κ-coefficient reveals whether the reproducibility of a diagnostic test exceeds that obtained by chance alone (Landis and Kock, 1977; Silcocks, 1983): $\kappa = 1$ means full agreement and $\kappa = 0$ is found when the agreement may solely be explained by chance; $\kappa < 0$ is found when the observed agreement is less than expected by chance; $\kappa > 0.70$ indicates a high degree of concordance. The Spearman rank correlation test and contingency test were used to calculate correlation between histological grading and ploidy.

### Results

The results of histological grading obtained after consensus are listed in Table I. According to the WHO classification system, 11.9% of the tumours were well differentiated, 32.2% were moderately differentiated and 55.9% were poorly differentiated. According to the Gleason classification system, 15.3% of the tumours had a Gleason score of 2–4, 69.5% Gleason score 5–7 and 15.3% Gleason score 8–10. There was no significant difference between the two outcome groups of patients with regard to the WHO ($P = 0.67$) or the Gleason ($P = 0.50$) classification systems. Eight per cent of the tumours in the therapy-sensitive group were well differentiated, compared with 16% in the therapy-resistant group. Furthermore, in the therapy-sensitive group 33% of the tumours were moderately and 59% were poorly differentiated compared with 31% and 53%, respectively, in the therapy-resistant group. Eleven per cent achieved Gleason score 2–4 in the therapy-sensitive group compared with 19% in the therapy-resistant group. Sixty-nine per cent of the patients have Gleason score 5–7. Seventy-four per cent of these were in the therapy-sensitive group, compared with 66% in the therapy-resistant group. The tumours with Gleason scores of 8–10 were equally distributed in the two groups. Thus, neither the Gleason system nor the WHO system discriminated between the two groups of patients.

The overall agreement, the agreement by chance, and the κ-values for the two pathologists are given in Table II with regard to both intra- and inter-observer reproducibility. κ-statistical evaluation revealed weak intra- and inter-observer reproducibility of both grading systems ($\kappa < 0.70$). The κ-values were low, even when the Gleason scores were reduced to three levels. Further analysis of intra-observer variability for Gleason scores showed that the two observers differed by only one point in 88% and 83% of the cases. For inter-observer variation difference of only one Gleason point was found in 74.5% of cases (average of two independent gradings).

The results of the DNA ploidy distributions are given in Table III. Overall 48% of tumours were diploid, 20% were tetraploid and 32% were aneuploid. In the therapy-sensitive group, 55% of the carcinomas were diploid, 15% were tetraploid and 30% of the carcinomas were aneuploid. In the therapy-resistant group of patients 41% of the carcinomas were diploid, 25% were tetraploid and 34% were aneuploid. There was no significant difference ($P = 0.38$) between the two outcome groups of patients with regard to diploid, tetraploid and aneuploid tumours. Thus, DNA ploidy of the primary prostate cancer could not discriminate between the good- and bad-outcome groups of patients. There is, furthermore, no correlation between DNA ploidy and histological grading (ploidy *vs* WHO, $P = 0.80$; ploidy *vs* Gleason, $P = 0.62$).

**Figure 4** Aneuploid range histogram of primary prostate adenocarcinomas with a prominent peak between 2c and 4c and several nuclei exceeding 5c.

**Figure 5** Diploid range histogram of primary prostate adenocarcinomas with two nuclei exceeding 5c.

### Discussion

Carcinoma of the prostate is a tumour with considerable biological variability. Even after metastases appear, survival varies considerably. Clinical stage and histological grade are accepted as important parameters for therapy decision and prediction of prognosis. There is a general agreement regarding the prognostic value of histological grading systems for carcinomas of the prostate gland, poorly differentiated carcinomas showing more aggressive behaviour than well-differentiated tumours (Broders, 1926; Whitmore, 1973; Gleason *et al.*, 1974; Mostofi, 1976). As the treatment decision for any individual prostatic cancer patient is influenced by histological grade, the accuracy as well as the reproducibility of different grading systems are of utmost importance (ten Kate *et al.*, 1986).

The present study involved 59 patients chosen from the 273 patients in the SPCG-2 study (Jørgensen *et al.*, 1993). The SPCG-2 study was designed to include only well-differentiated and moderately differentiated tumours. Two experienced pathologists reviewed the histological slides from these 59 patients independently, and 27% and 37% of the tumours were graded as poorly differentiated. The histological slides were also reviewed on two separate occasions. Finally, as a final control the two pathologists reviewed them together. At this consensus meeting, 56% of tumours were graded as poorly differentiated. Thus a weak inter- as well as intra-observer reproducibility was disclosed for both grading systems ($\kappa < 0.70$). When the κ-value for the traditional Gleason system was calculated, even worse inter- and intra-observer reproducibility was disclosed. The Gleason system is the sum of the two most dominant growth patterns, each

Table I Distribution of WHO and Gleason histological grades in the two outcome groups of patients, after Gleason scores (2–10) were reducd to three levels

| | Histological grading distribution | | | | | |
|---|---|---|---|---|---|---|
| | WHO | | | Gleason | | |
| | Therapy sensitive | Therapy resistant | | Therapy sensitive | Therapy resistant | |
| Level 1 | 2 (8%) | 5 (16%) | 7 | 3 (11%) | 6 (19%) | 9 |
| Level 2 | 9 (33%) | 10 (31%) | 19 | 20 (74%) | 21 (66%) | 41 |
| Level 3 | 16 (59%) | 17 (53%) | 33 | 4 (15%) | 5 (15%) | 9 |
| Total | 27 (100%) | 32 (100%) | 59 | 27 (100%) | 32 (100%) | 59 |

Table II The overall agreement, one- or two-level disagreement, agreement by chance and κ-values after Gleason were reduced to three levels. 'A' and 'B' are the two pathologists' intra-observer results. I and II are the inter-observer results after the two pathologists independently reviewed all the histological slides on two different occasions

| | Intra-observer | | | | Inter-observer | | | |
|---|---|---|---|---|---|---|---|---|
| | WHO | | Gleason | | WHO | | Gleason | |
| | A | B | A | B | I | II | I | II |
| Overall agreement | 0.70 | 0.61 | 0.80 | 0.78 | 0.66 | 0.71 | 0.75 | 0.70 |
| Disagree 1 level | 0.30 | 0.37 | 0.18 | 0.20 | 0.34 | 0.25 | 0.25 | 0.25 |
| Disagree >1 level | 0.00 | 0.02 | 0.02 | 0.02 | 0.00 | 0.04 | 0.00 | 0.05 |
| Agreement by chance | 0.45 | 0.43 | 0.60 | 0.51 | 0.46 | 0.41 | 0.56 | 0.54 |
| Kappa (κ) | 0.46 | 0.32 | 0.49 | 0.55 | 0.37 | 0.52 | 0.42 | 0.34 |

Table III DNA ploidy distributions in the two outcome groups of patients

| | DNA ploidy distributions | | |
|---|---|---|---|
| | Therapy sensitive | Therapy resistant | |
| Diploid | 15 (55%) | 13 (41%) | 28 |
| Tetraploid | 4 (15%) | 8 (25%) | 12 |
| Aneuploid | 8 (30%) | 11 (34%) | 19 |
| Total | 27 (100%) | 32 (100%) | 59 |

scored from 1 to 5. The Gleason sum therefore ranges from 2 to 10, and intra- and inter-observer variation may easily occur. Our results are in accordance with other reports (Mostofi, 1976; ten Kate *et al.*, 1986; Gleason, 1992; Swanholm *et al.*, 1992) and stress that histological grading is subjective and inaccurate. Thus, histological grade is not a reliable factor when used as an inclusion/exclusion criterion in clinical trials or as a parameter for treatment decision. Bearing in mind the low reproducibility, the results of our study indicate that histological grade is less important than usually anticipated.

The present study was designed in such a way that the clinical outcome differed significantly for the two groups of patients. The first group consisted of patients who experienced fast progression and death due to cancer despite endocrine ablation treatment. The second group consisted of patients who showed a good response to endocrine ablation treatment and a favourable prognosis. If there is any prognostic importance of histological grade for the individual patient with metastatic prostatic cancer, we should expect a high rate of poorly differentiated tumours or high Gleason scores in the poor-prognosis group. Likewise, those patients with well-differentiated tumours and low Gleason scores should be in the good-prognosis group. However, this was not the case.

According to the WHO classification, two of seven high-grade tumours were from patients in the good-prognosis group and the remaining five were from patients with a poor prognosis. Of the 33 poorly differentiated tumours, 16 were in the good-prognosis group compared with 17 in the poor-prognosis group. With respect to the Gleason system, nine patients had Gleason scores of 3 and 4, and of these three patients belonged to the good-prognosis group and six belonged to the poor-prognosis group. Nine patients had tumours with a Gleason score of 8 or 9, which are expected to be aggressive. Four of these belonged to the good-prognosis group and five to the poor-prognosis group. We were not able to find any prognostic value of the two common histological grading systems in patients with metastatic prostatic cancer. Similar conclusions have been reached in other studies in which several multivariate analyses of prognostic factors in metastatic prostate cancer have disclosed weak prognostic importance of histological grading (Emrich *et al.*, 1985; De Voogt *et al.*, 1989; Mulders *et al.*, 1990; Ernst *et al.*, 1991; Hedlund *et al.*, 1991). On the other hand, a similarly designed study (Miller *et al.*, 1991) found that 76% of poorly differentiated tumours occurred in a bad outcome group and 65% of the well-differentiated tumours occurred in a good-outcome group in patients with metastatic disease.

Because of the possibility of using paraffin-embedded archival tumour material for DNA ploidy analysis, patient groups with known clinical outcome can be selected for studies of the prognostic value of ploidy. A review of 47 different DNA ploidy studies involving 3493 patients has indicated that in most studies DNA aneuploidy is positively correlated with high-grade tumours, advanced stage and, consequently, with short time to progression and death (Visakorpi *et al.*, 1993). Most of these studies were performed on localised or locally advanced-stage disease. Only a few studies on metastatic (M1) disease have been reported. The present analysis of 59 cases did not demonstrate any significant difference ($P = 0.38$) between the two outcome groups of patients with regard to diploid, tetraploid and aneuploid tumours. Our results concur with other ploidy investigations on metastatic prostatic cancer (White *et al.*, 1990; Adolfsson and Tribukait, 1991; Hedlund *et al.*, 1991). When distant metastases have appeared, the prognostic importance of DNA ploidy seems to be low. Miller *et al.* (1991) also found overall the same distribution of diploid, tetraploid and aneuploid tumours as the present study. On the other hand, Miller *et al.* found that 64% of diploid tumours occurred in patients in a good-outcome group and 88% of non-diploid tumours in patients in a poor-outcome group, compared with 54% and 62%, respectively, in our study. Miller *et al.* concluded that DNA ploidy was a highly significant prognostic factor. One explanation for these divergent results may be that Miller *et al.* used stricter patient selection criteria. The patients in the poor-outcome group died during the first year, whereas those in the good-outcome group survived for more than 5 years. In general, less than 20% of patients survive more than 5 years after distant metastases have appeared (Blacard *et al.*, 1973), a fact that may explain the imbalance in the number of patients in the two outcome groups in the study by Miller *et al.* (1991). However, Miller *et al* found non-diploid tumours (36%) in the good-prognosis group and diploid tumours (12%) in the poor-prognosis group.

Some reports indicate that diploid and tetraploid tumours respond better to hormonal therapy than aneuploid tumours (Tavares *et al.*, 1973; Zetteberg and Esposti, 1980; Zetteberg and Forsslund, 1991). In these studies the endocrine treatment was initiated at earlier stages of the disease, where DNA ploidy has indicated important prognostic information for groups of patients. The present study confirms other reports (White *et al.*, 1990; Adolfsson and Tribukait, 1991; Hedlund *et al.*, 1991) that ploidy cannot predict the response to endocrine treatment in individual patients when distant metastases have already appeared.

When analysing tumour material from the prostate gland, heterogeneity within the tumour should be taken into account (Lange and Narayan, 1983). Most of our biopsies were obtained by TUR, only six by Tru-cut and two by open prostatectomy. TUR mostly samples tissue from transitional zone lobes and periuretheral glands (Villers *et al.*, 1991), and the tissue samples are dependent on how 'radical' is the resection performed. Seventy per cent of the patients had tumours of T category 3 or 4. These tumours may originate from the peripheral zone, and the cancer tissue obtained by TUR might be representative of the biological potential of the cancer tissue that has invaded the prostate capsule or periprostatic tissue. Even though there is no general consensus regarding the histogram analysis, and the histological material analysed might represent the most aggressive part of the tumour, a similar percentage of aneuploid tumours (Table III) was found in both patient groups.

To conclude, we could not find any significant differences between therapy-sensitive and therapy-resistant patients when using either histological grade or ploidy status evaluation. When distant metastases have appeared the prognostic importance of histological grade as well as DNA ploidy seems to be minor according to present results. Furthermore, histological grading is subjective and inaccurate. Future investigations should search for other prognostic factors that can predict more accurately the outcome in individual patients with metastatic prostatic cancer.

**Acknowledgements**

This work was supported by the Norwegian Cancer Society. The authors want to thank the following hospitals' departments of urology and pathology for their support in lending us histological sections and paraffin blocks. Norway: Central Hospital Akershus, Central Hospital Aust-Agder, Central Hospital Buskerud, Baerum Hospital, Central Hospital Molde, Central Hospital Rogaland, Central Hospital Sogn og Fjordane, Central Hospital Telemark, Ullevål Hospital, Laboratorium for Patologi, Oslo, Central Hospital Ålesund. Sweden: Boden Hospital, Sandviken Hospital, Central Hospital Växsjö. We also acknowledge Ruth Puntervold for her skilful technical assistance and Olav Kaalhus and Magne Bryne for statistical support. We thank Dr José Lopes and Dr Jahn M Nesland for fruitful discussions.

**References**

ADOLFSON J AND TRIBUKAIT B. (1991). Modal DNA-values in prostate cancers patients with deferred therapy or endocrine therapy. *Acta Oncol.*, **30**, 209-210.

BERNER AA, DANIELSEN HE, PETTERSEN EO, FOSSÅ SD, REITH A AND NESLAND JM. (1993). DNA distribution in prostate. Normal glad, benign and premalignant lesions, and subsequent adenocarcinomas. *Anal. Quant. Cytol.*, **15**, 247-252.

BLACARD CE, BYAR DP AND JORDAN WP. (1973). Orchiectomy for advanced prostatic carcinoma. A reevaluation. *Urology*, **1**, 553-560.

BRODERS AC. (1926). Grading and practical application. *Arch. Pathol. Lab. Med.*, **68**, 376-381.

DE VOOGT HJ, SUCIU S AND SYLVESTER R. (1989). Multivariate analysis of prognostic factors in patients with advanced prostatic cancer. *J. Urol.*, **141**, 883-888.

EMRICH LJ, PRIORE RL, MURPHY GP, BRADY MF AND THE INVESTIGATORS OF THE NATIONAL PROSTATIC CANCER PROJECT. (1985). Prognostic factors in patients with advanced stage prostate cancer. *Cancer Res.*, **45**, 5173-5179.

ERNST DS, HANSON J, VENNER PM AND THE URO-ONCOLOGY GROUP OF NORTHERN ALBERTA. (1991). Analysis of prognostic factors in men with metastatic prostate cancer. *J. Urol.*, **146**, 372-376.

FORSSLUND G, ESPOSTI PL, NILSSON B AND ZETTERBERG A. (1992). The prognostic significance of nuclear DNA content in prostatic carcinoma. *Cancer*, **69**, 1432-1439.

FRANKFURT OS, CHIN JL, ENGLANDER LS, GRECO WR, PONTES JE AND RUSTUM YM. (1985). Relationship between DNA ploidy, glandular differentiation, and tumor spread in human prostate cancer. *Cancer Res.*, **45**, 1418-1423.

GLEASON DF. (1992). Histologic grading of prostate cancer. *Hum. Pathol.*, **23**, 273-279.

GLEASON DF AND THE VETERANS ADMINISTRATION COOPERATIVE UROLOGICAL RESEARCH GROUP. (1974). Prediction of prognosis for prostatic adenocarcinoma by combined histological grading and clinical staging. *J. Urol.*, **111**, 58-64.

GLEASON DF AND THE VETERANS ADMINISTRATION COOPERATIVE UROLOGICAL RESEARCH GROUP. (1977). Histologic grading and clinical staging of prostatic carcinoma. In *Urologic Pathology: The Prostate*, Tannenbaum M (ed.) pp. 171-197. Lea & Febiger: Philadelphia.

HEDLUND PO, ESPOSTI P, FALKMER U AND JACOBSSON H. (1991). DNA as prognostic marker in advanced high-grade prostatic cancer. *Acta Oncol.*, **30**, 215-217.

JACOBS SC. (1983). Spread of prostatic cancer to bone. *Urology*, **21**, 337-344.

JØRGENSEN T, TVETER K, MEMBERS OF SPCG-2 GROUP AND JØRGENSEN L. (1993). Total androgen suppression in metastatic prostatic cancer: Experience from Scandinavian prostatic cancer group study no. 2. *Eur. Urol.*, **24**, 466-470.

LANDIS JR AND KOCK GG. (1977). The measurement of observer agreement for categorical data. *Biometrics*, **33**, 159-174.

LANGE PH AND NARAYAN P. (1983). Understaging and undergrading of prostate cancer. Argument for postoperative radiation as adjuvant therapy. *Urology*, **21**, 113-118.

LEE SE, CURRIN SM, PAULSON DF AND WALTHER PJ. (1988). Flow cytometric determination of ploidy in prostatic adenocarcinoma. A comparison with seminal vesicle involvement and histopathological grading as a predictor of clinical recurrence. *J. Urol.*, **140**, 769-774.

MAHLER C AND DENIS L. (1992). Management of relapsing disease in prostate cancer. *Cancer*, **70**, 329-334.

MERKEL DE AND McGUIRE WL. (1990). Ploidy, proliferative activity and prognosis. DNA flow cytometry of solid tumours. *Cancer*, **65**, 1194-1205.

MILLER J, HORSFALL DJ, MARSHALL VR, RAOE DM AND LEONG ASY. (1991). The prognostic value of deoxyribonucleic acid flow cytometric analysis in stage D2 prostatic carcinoma. *J. Urol.*, **145**, 1192-1196.

MOSTOFI FK, SESTERHENN I AND SOBIN LH. (1980). *International Histological Classification of Tumours*, No. 22. World Health Organization: Geneva.

MOSTOFI FK. (1976). Problems of grading carcinoma of prostate. *Semin. Oncol.*, **3**, 161-169.

MULDERS PFA, DIJKMAN GA, DEL MORAL PF, THEEUWES AGM, DEBRUYNE FMJ AND MEMBERS OF THE DUTCH SOUTHEASTERN UROLOGICAL COOPERATIVE GROUP. (1990). Analysis of prognostic factors in disseminated prostatic cancer. *Cancer*, **65**, 2758-2761.

PETERS JM, MILES BJ, KUBUS JJ AND CRISSMAN JD. (1990). Prognostic significance of nuclear DNA content in localized prostatic adenocarcinoma. *Anal. Quant. Cytol. Histol.*, **12**, 359-365.

SILCOCKS PBS. (1983). Measuring repeatability and validity of histological diagnosis – a brief review with some practical examples. *J. Clin. Pathol.*, **36**, 1269-1275.

SWANHOLM H, STARKLINT H, GUNDERSEN HJG, FABRICIUS J, BARLEBO H & OLSEN S. (1989). Reproducibility of histomorphologic diagnosis with special reference to the kappa statistic. *APMIS*, **97**, 689-698.

SWANHOLM H, STARKLINT H, BARLEBO H AND OLSEN S. (1990). Histological evaluation of prostatic cancer (II): Reproducibility of a histological grading system. *APMIS*, **98**, 229-236.

TAVARES AS AND COSTA MJ. (1973). Correlation between ploidy and progression in prostatic carcinoma. *J. Urol.*, **109**, 676–679.

TEN KATE FJW, GALLEE MPW, SCHMITZ PIM, JOEBSIS AC, VAN DER HEUL RO, PRINS ME AND BLOM JHM. (1986). Problems in grading of prostatic carcinoma. *World J. Urol.*, **4**, 147–152.

VILLERS AA, MCNEAL JE, FREIHA FS AND STAMEY TA. (1990). Development of prostatic carcinoma. Morphometric and pathologic features of early stages. *Acta Oncol.*, **30**, 145–151.

VISAKORPI T, KALLIONIEMI OP, KOIVULLA T AND ISOLA J. (1993). New prognostic factors in prostatic carcinoma. *Eur. Urol.*, **24**, 438–449.

WHITE RW, DE VERE DEITCH AD, TESLUK H, LAMBORN KR AND MEYERS FJ. (1990). Prognosis in disseminated prostate cancer as related to tumor ploidy and differentiation. *World J. Urol.*, **8**, 47–50.

WHITMORE WF. (1973). The natural history of prostate cancer. *Cancer*, **32**, 1104–1112.

WILLIAMS NN AND DALY JM. (1990). Flow cytometri and prognostic implications in patients with solid tumors. *Surg. Gynecol. Obstet.*, **171**, 257–266.

ZETTEBERG A AND ESPOSTI PL. (1980). Prognostic significance of nuclear DNA levels in prostatic carcinoma. *Scand. J. Urol. Nephrol.*, **55** (Suppl.), 53–58.

ZETTEBERG A AND FORSSLUND G. (1991). Ploidy level and tumor progression in prostatic carcinoma. *Acta Oncol.*, **30**, 193–199.